# Zirconium Coordination Chemistry and Its Role in Optimizing Hydroxymate Chelation: Insights from Molecular Dynamics

**DOI:** 10.1021/acsomega.3c04083

**Published:** 2023-09-19

**Authors:** Giulia Sormani, Aruna Korde, Alex Rodriguez, Melissa Denecke, Ali Hassanali

**Affiliations:** † The “Abdus Salam” International Centre for Theoretical Physics, I-34151 Trieste, Italy; ‡ International Atomic Energy Agency, A-1400 Vienna, Austria; ¶ Dipartimento di Matematica e Geoscienze, University of Trieste, 34127 Trieste, Italy

## Abstract

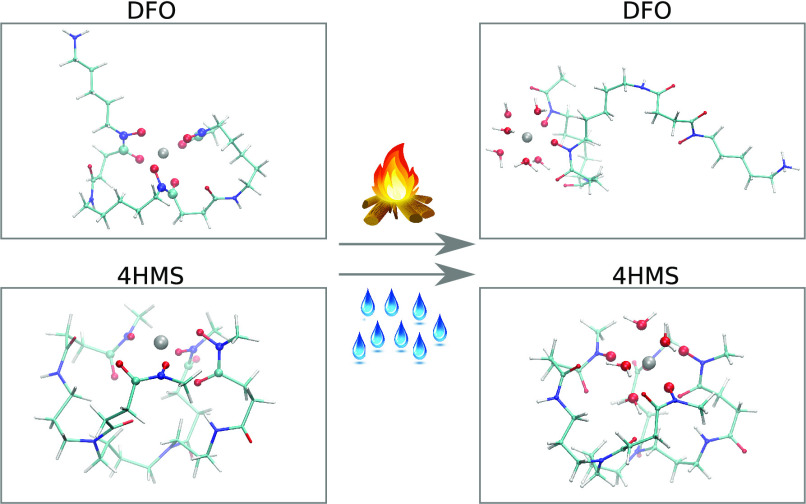

In the past decade, there has been a growth in using Zirconium-89 (^89^Zr) as a radionuclide in nuclear medicine for cancer diagnostic imaging and drug discovery processes. Although one of the most popular chelators for ^89^Zr, desferrioxamine (DFO) is typically presented as a hexadentate ligand, our work suggests a different scenario. The coordination structure of the Zr^4+^–DFO complex has primarily been informed by DFT-based calculations, which typically ignore temperature and therefore entropic and dynamic solvent effects. In this work, free energy calculations using molecular dynamics simulations, where the conformational fluctuations of both the ligand and the solvent are explicitly included, are used to compare the binding of Zr^4+^ cations with two different chelators, DFO and 4HMS, the latter of which is an octadentate ligand that has been recently proposed as a better chelator due to the presence of four hydroxymate groups. We find that thermally induced disorder leads to an open hexadentate chelate structure of the Zr^4+^–DFO complex, leaving the Zr^4+^ metal exposed to the solvent. A stable coordination of Zr^4+^ with 4HMS, however, is formed by involving both hydroxamate groups and water molecules in a more closely packed structure.

## Introduction

Radionuclides continue to be added to the toolbox of nuclear medicine, which represents an important field of development for drug discovery and nuclear medicine.^1^ Presently, fluorine-18(^18^F) and gallium-68(^68^Ga) are the most used positron emission tomography (PET) radionuclides for molecular imaging studies in nuclear medicine.^2^ PET permits noninvasive localization and quantification with relatively small amounts of radioactivity due to its high inherent sensitivity. The short half-lives of both these radionuclides (110 and 68 min, respectively), however, pose limitations for imaging biochemical processes with slow pharmacokinetics, such as localization of macromolecules at targeted tissues in macromolecular-based (e.g., antibodies) cancer therapies.^3^ On the other hand, Zirconium-89 (^89^Zr) is suitable for radiolabeling and in vivo visualization of such processes due to its relatively long half-life (78.4h), which matches the slow pharmacokinetics of macromolecular therapeutics.^4^

Most emerging therapeutics are based on macromolecules rather than small organic moieties, especially macromolecules that specifically target cancer cells through monoclonal antibodies, cell tracking agents, nucleotides, and nanoparticle systems.^5^ Nearly 25 clinical studies are currently ongoing with ^89^Zr-labeled molecules.^6^ Zirconium-89 can be produced in medium energy medical cyclotrons through the ^89^Y(p,n) ^89^Zr nuclear reaction.^7^ An ongoing International Atomic Energy Agency (IAEA) coordinated research project focuses on standardization of ^89^Zr production procedures and quality control.^8^ Designing a radiopharmaceutical to transfer the radionuclide (such as ^89^Zr) to specific cells and retain it there for a desired time, with minimum accumulation and faster clearance from other nontarget tissues, is a challenging process.

Chelating molecules in radiopharmaceutical design play an important role as anchors for radionuclide metals and for targeting vector macromolecules. In the case of ^89^Zr, the bacteria-produced siderophore desferrioxamine B (DFO) is mostly used for radiolabeling.^9,10^ DFO is an open-chain hexadentate chelate molecule having three hydroxamate groups as radiometal binding moieties and a terminal primary amine that allows conjugation with vector biomolecules. In 1964, Baroncelli et al. found a high affinity of Zr^4+^ ions towards hydroxamic acid groups, and in 1992, Meijs et al. reported successful radiolabeling of DFO with ^89^Zr and good in vitro stability of the resulting complex.^11^ Since then, many other DFO analogues^12^ and other chelators including acyclic and cyclic polyazacarboxylates^13,14^ have been studied in search of complexes which are more stable in vivo and avoid unspecific uptake of ^89^Zr in, for example, the bones.^15,16^ More recently over the past decade, the speciation and thermodynamic stability of Zr^4+^–DFO complexes in solution has been investigated through potentiometric, spectrophotometric, and mass spectrometry measurements.^17−20^ These studies have pointed to the possibility of the formation of non-mononuclear complexes involving DFO and Zr^4+^.

To streamline and guide such trial-and-error optimization efforts for chelator design, computational methods offer insight into the structure and thermodynamic stability of different chelates under varying conditions, mimicking complex biological systems. In this regard, several computational studies have been conducted in order to understand the chelating mechanism of DFO. Specifically, the coordination structure of Zr^4+^ with DFO has been discussed as an important parameter in possibly controlling its stability in vivo. Quantum chemistry-based calculations using density functional theory (DFT), for example, have shown that seven-^21^ or eight-coordinated^17,22^ complexes, with Zr^4+^ bound to the six oxygen atoms of DFO and to one or two oxygen atoms from surrounding water molecules, are found to be the most stable complexes. While DFT approaches are more accurate insofar as including electronic effects, the vast majority of theoretical studies of DFO and Zr^4+^ are conducted at 0 K, which neglect thermal effects involving fluctuations in the chelator and surrounding solvent molecules.

**Figure 1:**
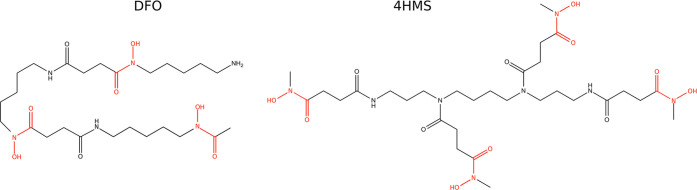
Schematic representation of DFO and 4HMS chelators. Hydroxamate groups of both molecules are highlighted in red.

In this work, we investigate the stability of two different Zr^4+^–chelator complexes in aqueous solution by means of molecular dynamics (MD) simulations which allow for a realistic sampling of both the conformational fluctuations of the chelator and the solvent environment. Specifically, the MD simulations allow for exploring the thermodynamics of the chelating complex, where the solvent and temperature effects are included in a realistic manner. The two considered chelators are deferoxamine-B (DFO)^23^ and 4HMS^24^ (N1,N5,N10,N14-tetra(*N*-hydroxy-*N*-methyl-1,4-dioxo-5-azapentyl)-1,5,10,14-tetraazatetradecane), which have recently been proposed in the literature^24^ as a better chelator of Zr^4+^ due to the presence of eight coordination sites.^24^ DFO and 4HMS are schematically shown in the left and right panels of Figure 1. The metal binding ability of these two chelators is heavily influenced by their hydroxamate groups, highlighted in red in Figure 1. DFO has three hydroxamate groups allowing, in principle, six oxygen atoms for coordination with Zr^4+^. On the other hand, 4HMS has four hydroxamate groups, increasing the possible coordination with Zr^4+^ to eight.

## Results

### Free Energy Landscape of Zr^4+^–DFO Complex in Solvent

We first performed a 1 μ*s* MD simulation of Zr^4+^–DFO complex in water at room temperature, choosing as the initial configuration, the DFT-optimized structure from ref (22) with 6-fold coordination. This structure was then solvated with over 3000 water molecules and two chloride ions are added to neutralize the system. This system is then subject to energy minimization. Besides the 6-fold coordination involving the hydroxamate groups, two coordinating trans-oriented oxygen atoms from water molecules were found in our optimized structure consistent with the findings of Holland and co-workers.^22^ Starting from this minimized structure, we initiated molecular dynamics simulations at 300 K. In order to track the dynamic evolution of the hydroxamate groups coordinating the Zr^4+^, we built a variable, CN_ligand_, mathematically defined in eq 2 in the Methods section, which essentially counts the number of oxygen atoms belonging to the DFO hydroxamate groups that are coordinated to Zr^4+^. Note that CN_ligand_ does not include oxygen atoms arising from coordinating water molecules which will be addressed separately. In this way, the CN_ligand_ can be used to quantify changes in the contribution of the hydroxamate groups to the first coordination sphere of the Zr^4+^ cation over the course of the molecular dynamics simulation.

**Figure 2:**
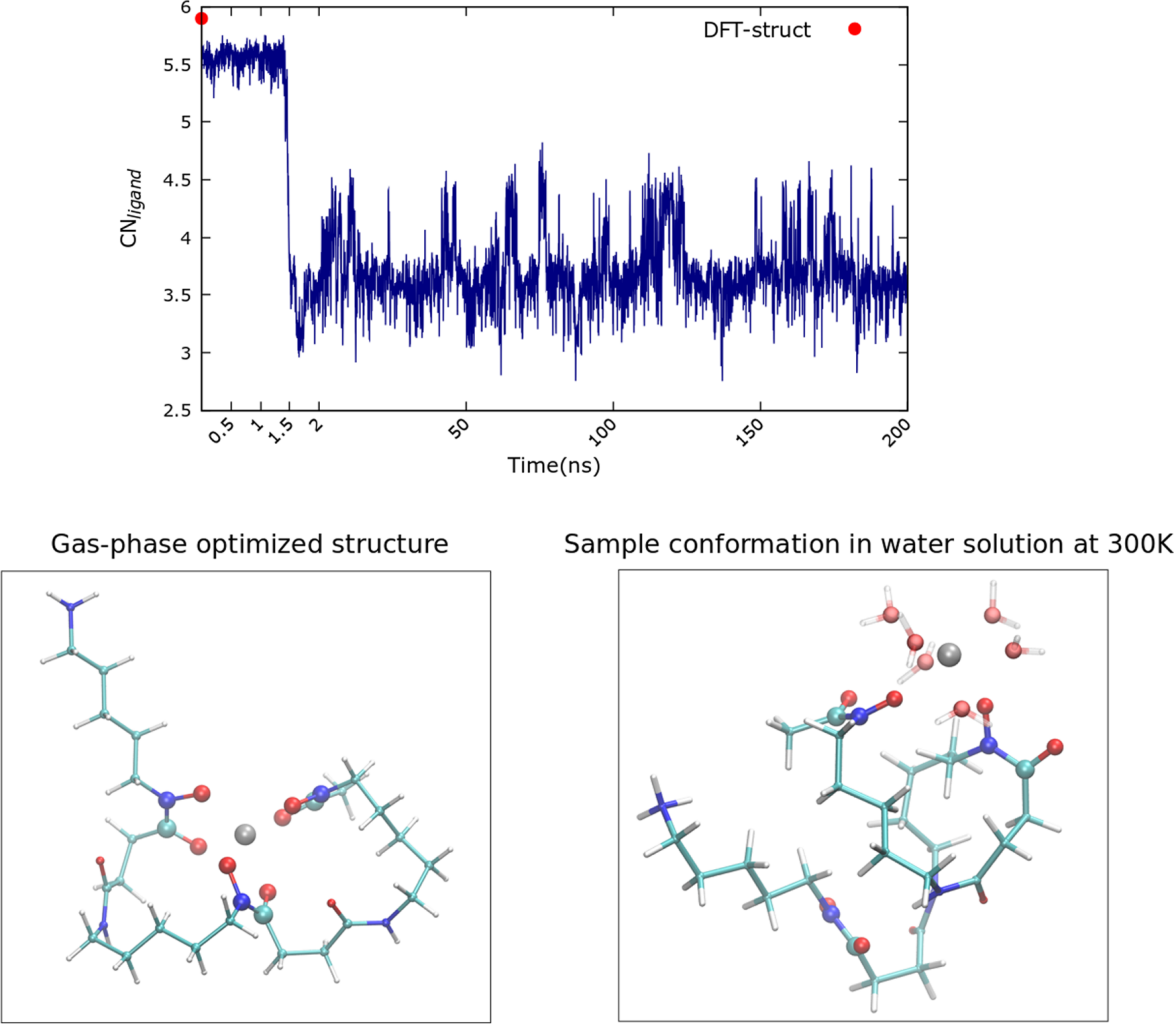
Upper panel: Temporal evolution of the CN_ligand_ collective variable along the first 200 ns of the simulation of the Zr^4+^–DFO complex in water. Bottom-left panel: DFT structure of the Zr^4+^–DFO complex from ref (22). Bottom-right panel: A snapshot of the simulation after 200 ns, water molecules in the first solvation shell of Zr^4+^ are depicted. For both bottom panels, the highlighted atoms of the DFO are those belonging to the hydroxamate groups.

The top panel of Figure 2 shows the temporal evolution of the coordination number (CN_*l*igand_) for the first 200 ns of the simulation. In the DFT-optimized structure, for example, the CN_ligand_ is ≃6. The bottom-left panel of Figure 2 shows that in this structure Zr^4+^ is surrounded by the three hydroxamate groups in the stable complex. At the beginning of the simulation, CN_ligand_ oscillates around a value of 5.5. This initial value differs by 0.5 from the coordination observed in the DFT structure as a consequence of small modifications of the DFO structure occurring during the equilibration phase of the system (see the Methods Section). One might interpret this as a fluctuation between bidentate and monodentate binding of the hydroxamate oxygen atoms to the Zr^4+^ cation.

The time series of the CN_ligand_ (upper panel of Figure 2) shows that there is a transition from CN_ligand_ ≃ 5.5 to CN_ligand_ ≃ 3.5 at ∼2 ns. The backward transition was not observed for the rest of the simulation lasting approximately 200 ns. The bottom-right panel of Figure 2 represents the snapshot of the simulation at 200 ns. The structure of the complex indicates a smaller number of DFO oxygen atoms involved in Zr^4+^ complexation. The fact that the hydroxamate group closest to the NH_3_ terminus has moved away from the inner Zr^4+^ coordination sphere suggests that, although the initial configuration (DFT-optimized structure) is likely to be the most stable structure on the potential energy landscape relevant at 0 K, its stability is altered by both temperature and water.

We turned to construct the free energy landscape of the Zr^4+^–DFO complex to better understand the results in Figure 2, where we observe no events involving the initial structure being revisited on the time scale of hundreds of nanoseconds. Therefore, one cannot reliably construct the correct Boltzmann weighted probabilities of the configurations in order to determine relative free energies of different conformational states. To circumvent this problem, we focused on examining the free energy profile along the CN_ligand_ using the umbrella sampling technique.^25^ The principle behind this method is that one adds an external potential to the system in order to achieve a non-Boltzmann sampling of regions that are poorly explored which can be subsequently reweighted to determine the correct free energy of the system.^25−27^

In detail, we performed a series of 1 μs long MD simulations of the Zr^4+^–DFO complex in water in which a harmonic biasing potential is put on the CN_ligand_ at different values. In total, we generated approximately 9 μs simulations for our analysis. All the simulations have the same value of the harmonic constant (*k* = 20*k*_B_*T*) but differ in the value of the center of the harmonic potential (centers go from CN_ligand_ = 2 to CN_ligand_ = 6, with a 0.5 step). This means that in each simulation, we are forcing (biasing) a specific number of coordinating hydroxamate oxygen atoms, given by the value of the center of the bias. The free energy surface (FES) is then reconstructed from the probability distribution of the CN_ligand_ obtained from each of these biased simulations using the WHAM method^28,29^ (code from ref (30)).

**Figure 3:**
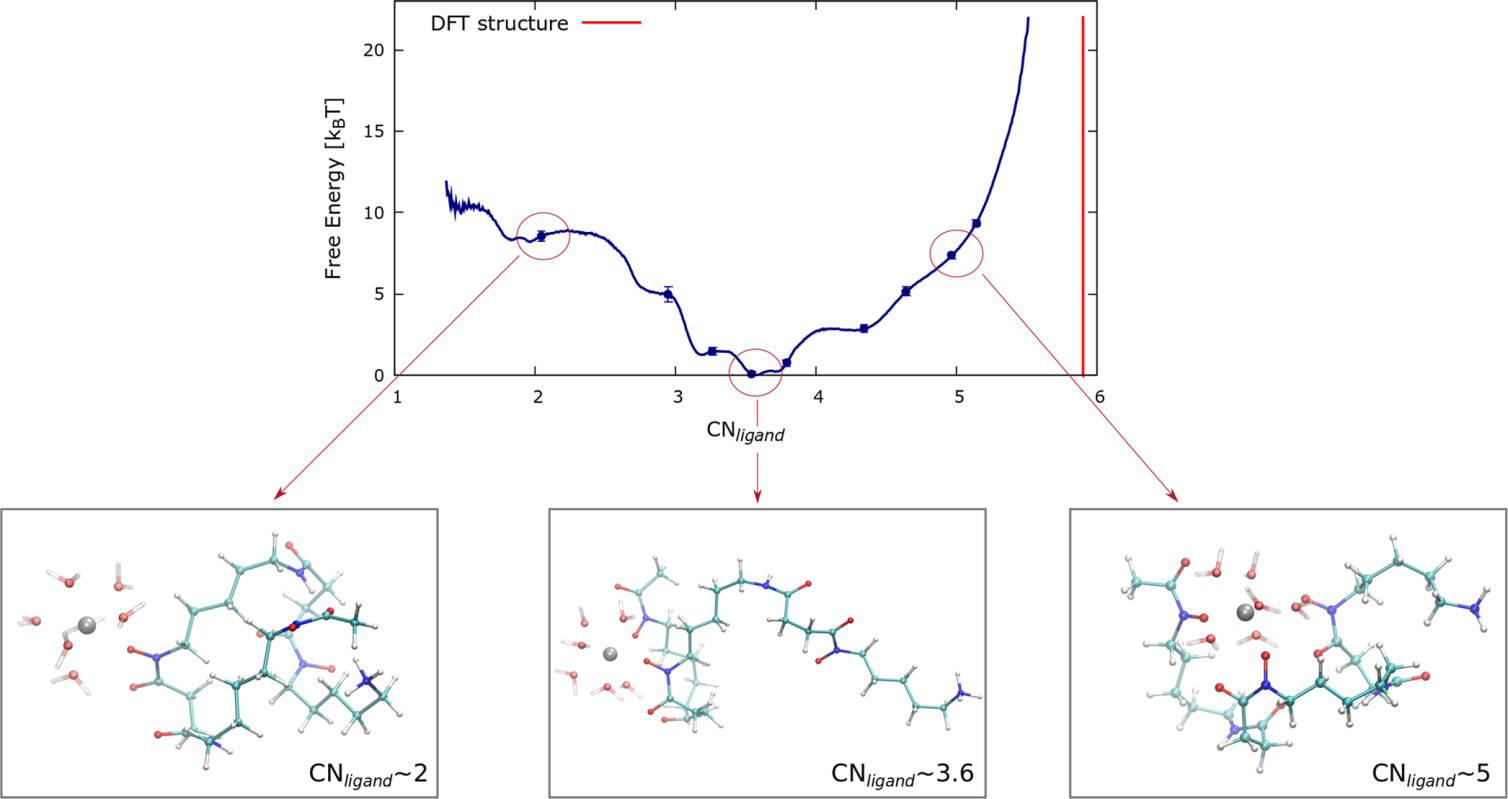
Upper panel: Free energy landscape of the Zr^4+^–DFO complex in water as a function of the CN_ligand_. The red line shows the magnitude of the CN_ligand_ for the DFT-optimized structure from ref (22). Lower panels: Representative conformations of the same complex, corresponding to different CN_ligand_ values. Water molecules belonging to the first solvation shell of Zr^4+^ are depicted; a more quantitative analysis of Zr^4+^ hydration will be presented in Figure 7.

The top panel of Figure 3 shows the FES obtained for the Zr^4+^–DFO complex along the CN_ligand_ coordinate. As a visual guide for the reader, the solid red vertical line shows the magnitude of CN_ligand_ obtained from our initial optimized structure. Also shown are error bars in the FES constructed using the Monte Carlo bootstrap method^31^ as implemented in ref (30). Rather strikingly, we observe that the DFT-optimized structure does not correspond to the global free energy minimum but is rather high up in energy, indicating that at room temperature, the 6-fold coordination in DFO is highly unstable by at least 20*k*_B_*T*. The global minimum of the FES is observed at CN_ligand_ ∼ 3.5, meaning that there are three to four oxygen atoms of DFO coordinated to Zr^4+^.

The bottom panels of Figure 3 visually depict representative snapshots obtained along different regions of the FES. The central structure corresponds to CN_ligand_ ≃ 3.6 (the global minimum of the FES) where only two of the three hydroxamate functional groups of DFO interact with Zr^4+^. This illustrates that the rest of the molecule involving the N-terminus is not involved in the chelation complex. The right-most structure of Figure 3 corresponds to CN_ligand_ ≃ 5 with the three hydroxamate groups surrounding the Zr^4+^ cation as in the DFT structure. This complex is higher up in free energy. As we will see below, finite temperature alters the conformational fluctuations of the DFO molecule as well as the hydrogen bonds between the hydroxamate groups and the water molecules which in turn affect the binding mechanisms.

In our simulations, we do not find any specific binding of the two chloride ions to Zr^4+^. The smallest distance that the Cl^–^ ions approach the Zr^4+^ is 5 Å and this happens only very transiently. As usual, this is a consequence of a subtle balance between entropic and enthalpic driving forces. Entropy naturally favors the Cl^–^ ions to be far from Zr^4+^. On the other hand, strong interactions between the ions and metal can switch the balance as it does for DFO. Since the Zr^4+^ ion is bound to the DFO hydroxymate groups and also fully solvated, any electrostatic interaction between the Zr^4+^ and Cl^–^ ions is essentially screened. Furthermore, there are no other strong interactions that can form between the metal and Cl^–^ ions.

### Potential Energy Landscape of the Zr^4+^–DFO Complex

As alluded earlier, several studies using DFT-based optimizations showed that the most stable 1:1 Zr^4+^–DFO complex is with six oxygen atoms stemming from the DFO molecule coordinated to Zr^4+^.^17,21,22^ Our results however, paint a different picture when temperature and solvent are explicitly included. On the one hand, there have been numerous studies of molecular systems (regarding, for example, the binding between ligands and proteins), showing that both solvent and temperature can have a drastic effect on both the binding mechanisms and subsequently thermodynamics.^32−38^ Specific solvation of water in response to the presence of a solute such as a hydrophobic moiety or a dipole or charge redistribution is known to play a critical role in a wide range of physical and chemical processes.^39−42^ At the same time, we did not explicitly include electronic effects in our model.

**Figure 4:**
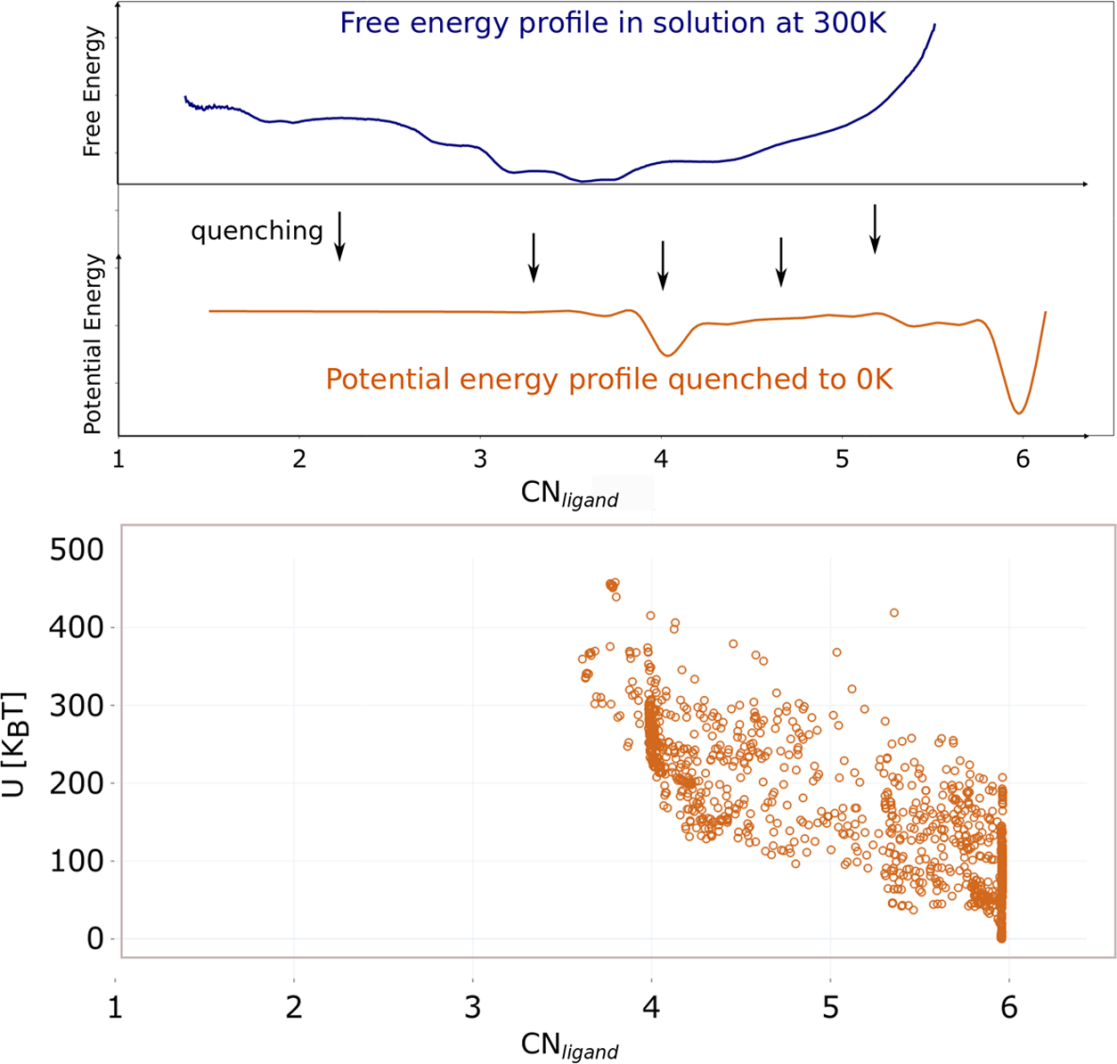
Top panel: Schematic representation of the geometry optimization procedure. Many initial configurations are taken from different regions of the free energy landscape, quenching them at 0 K, the resulting potential energy landscape has a completely different profile. Bottom panel: Potential energy (U) vs CN_ligand_ for all optimized conformations of the Zr^4+^–DFO complex.

In order to understand the origins of these effects better and to explore the topography of the underlying potential energy landscape (PES), we conducted an inherent structure analysis.^43,44^ This procedure has first been used to study the potential energy landscape of liquids^44−46^ and soft-matter systems such as proteins.^47−49^ The top panel of Figure 4 shows a schematic of how this process is conducted for our specific system. Here, we performed a geometry optimization of many structures sampled from the FES at 300 K (top panel of Figure 4) that start from different initial values of the CN_ligand_. These configurations are subsequently quenched via geometry optimization on the PES (middle panel of Figure 4).

To allow for a more direct comparison with the conditions in previous DFT simulations, all of the water molecules are removed and then the total potential energy (*U*) is optimized. The optimization is performed by the steepest descent method^50,51^ followed by the application of the conjugate gradient algorithm.^52^ The combination of these two techniques allows for identification of the nearest minimum of *U* for each starting structure. A threshold of 10 × 10^–5^*k*_B_*T*/Å is used for convergence of the forces.

The bottom panel of Figure 4 shows a scatter plot of *U* vs CN_ligand_ obtained for all of the optimized structures. We observe that the optimized geometries on the PES localize to two regions corresponding to CN_ligand_ ≃ 4 and CN_ligand_ ≃ 6. The global minimum of *U* is for CN_ligand_ ≃ 6, which is several 100*k*_B_*T* lower than the other coordination configurations. These results are fully consistent with previously reported DFT calculations^22^ and confirm that the differences we observe between the PES and FES arise from temperature-induced conformational disorder and solvent effects. Furthermore, the agreement with DFT suggests that our interaction potential is sufficiently accurate to capture the structure of the chelator complex.

### Comparing Free Energy Landscape of DFO and 4HMS

In a recent study, Alnahwi and co-workers showed that the 4HMS chelator presented improved chelating properties.^24^ Specifically, they found using both in vitro and in vivo studies that the molar activity of 4HMS complexed with Zr^4+^ is at least three times higher than that of DFO. In addition, by performing DFT calculations, they demonstrated that all of the eight oxygen atoms belonging to the four hydroxamate groups are bound to the Zr^4+^ with a distance less than 2.4 Å.

**Figure 5:**
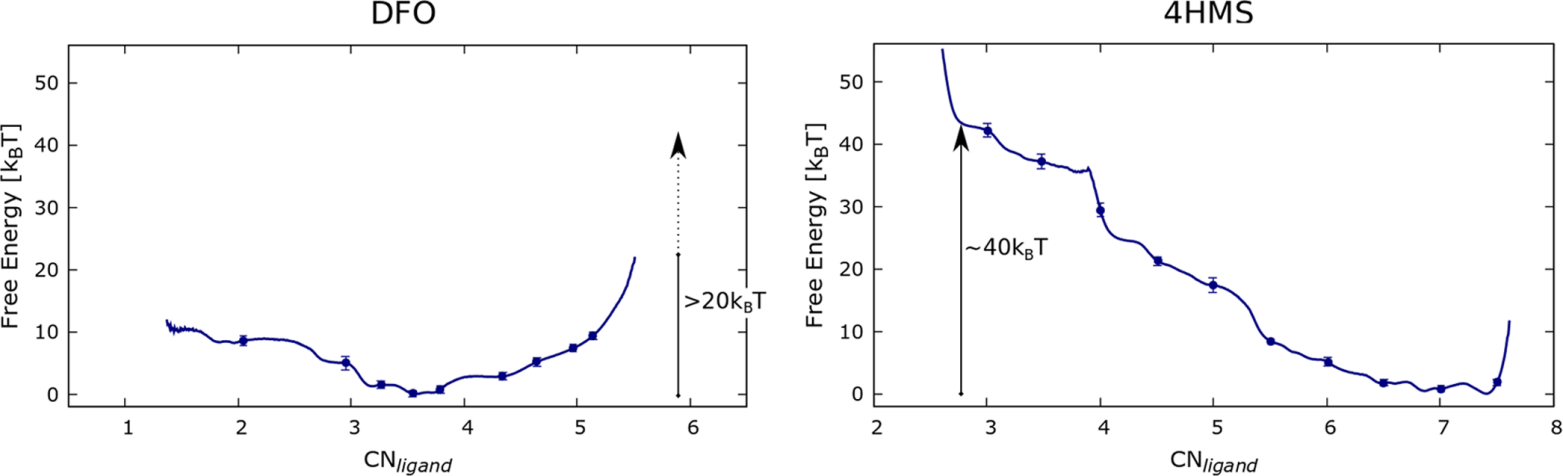
Left panel: Free energy profile of the Zr^4+^–DFO complex in solvent as a function of the CN_*ligand*_ collective variable. Right panel: The same for the Zr^4+^–4HMS complex.

In order to compare the free energy landscapes for DFO and 4HMS, we repeated our umbrella sampling simulations of 4HMS using again the CN_ligand_ as a collective variable. The harmonic constant chosen was *k* = 20*k*_B_*T* with the centers of the constraints going from CN_ligand_ = 2 to CN_ligand_ = 8 using a step of size of 0.5. The total accumulated length of these MD simulations was 11 μs. Figure 5 compares the FES of the Zr^4+^–DFO complex (left) with that of Zr^4+^–4HMS (right panel). The FES for the two systems is strikingly different and confirms the experimental observations of the greater efficacy of 4HMS as a chelator compared to DFO. Specifically, the free energy minimum of 4HMS corresponds to a structure where all four hydroxamate groups envelop the Zr^4+^. For example, configurations at CN_ligand_ ≃ 3.5 which is the most stable value for DFO, are now ≃35*k*_B_*T* higher in free energy than the global minimum in 4HMS. Thus, in sharp contrast to what is observed for DFO, higher CN_ligand_ are more favored than lower ones in agreement with the with the experimental observations.^24^

Figure 5 shows that the most stable Zr^4+^–DFO complexes are located at low values of CN_ligand_ (∼3.5), while for 4HMS, these are located at high values of CN_ligand_ (≥7). Looking at the free energy profile of DFO, we observe that in order to create a coordination sphere where the three hydroxymate groups of the DFO fully coordinate the ion (with CN_ligand_ values close to ∼6.0), there is a free energy cost of at least 20*k*_B_*T*. On the other hand in the Zr^4+^–4HMS complex, the stable structure with the lowest free energy is the one where the four hydroxymate groups encapsulate the Zr^4+^ ion. In this case, reducing the coordination to a value similar to the location of the free energy minimum in DFO requires a free energy cost (penalty) of ∼35*k*_B_*T*. As we will see later, the inclusion of water molecules explicitly into the free energy landscape does not change these conclusions.

#### Conformational Heterogeneity: DFO versus 4HMS

**Figure 6:**
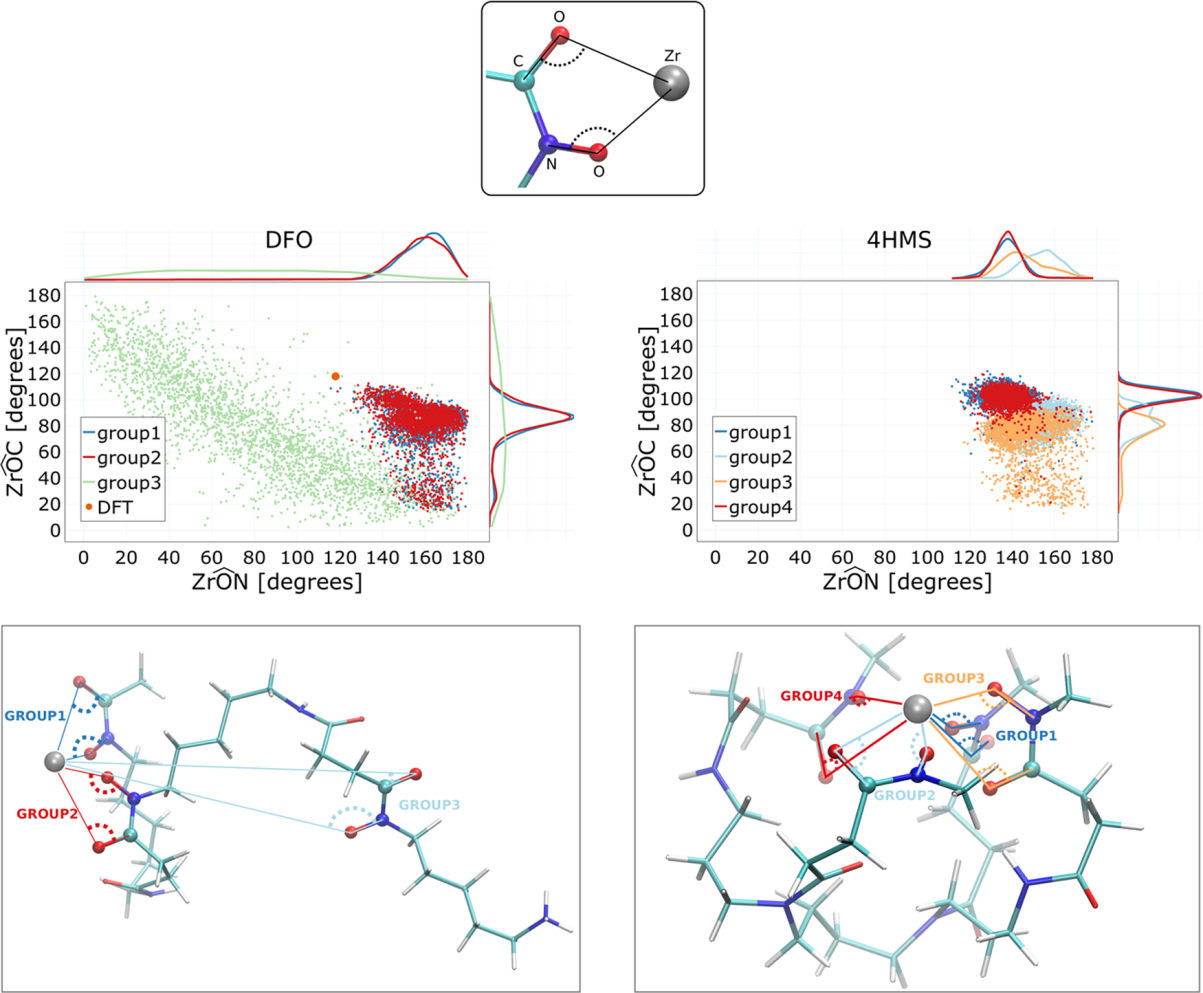
Top-left panel (DFO): Scatter plot of the Zr^4+^–O–N and Zr^4+^–O–C angles (O, N, and C atoms belonging to the three hydroxamate groups of DFO). The analyzed conformations belong to the 1 μs trajectory at the free energy minimum (center of the bias at CN_ligand_ = 3.5). Top-right panel (4HMS) shows the same scatter plot but for Zr^4+^–O–N and Zr^4+^–O–C angles of the four 4HMS hydroxamate groups. The conformation belongs to the 1 μs trajectory with the center of the bias at CN_ligand_ = 7.5. Lower panels: the angles considered are highlighted in snapshots of the analyzed trajectories.

The preceding results indicate that there are some important differences in the manner in which the hydroxamate groups of DFO and 4HMS envelope the Zr^4+^ ion. In order to quantify these differences, we examined the orientation of the hydroxamate groups relative to the Zr^4+^ ion, specifically in the free energy minima of DFO and 4HMS. For both systems, we considered two angles for each hydroxamate group: one formed by Zr^4+^–oxygen–nitrogen and another formed by Zr^4+^–oxygen–carbon as depicted in the top panel of Figure 6. In DFO (bottom-left panel of Figure 6), there are three groups correspondingly color-coded with each group yielding two angles while in 4HMS (bottom-right panel of Figure 6) there are four groups.

The top left and top right panels of Figure 6 show scatter plots of the two angles for DFO and 4HMS, respectively, obtained from independent configurations sampled along the trajectory at the position of the free energy minimum. The colors correspond to different hydroxamate groups (group1–group3 for DFO and group1–group4 for 4HMS). Above and on the right side of each scatter plot, the probability distributions of the corresponding angles are also shown. In the case of DFO, we see that the angular distributions of group1 and group2 (the ones interacting with Zr^4+^) are similar. For both groups, the peaks of the probability distributions are located at ≃160° for Zr^4+^–O–C angles and at ≃90° for Zr^4+^–O–C angles. Note that the peak positions do not correspond to the angles of the DFT-optimized structure (shown as solid green circles in the left panel). On the other hand for group3 which does not interact directly with Zr^4+^, the distributions are flat highlighting that this part of the DFO molecule has no preferred group orientation. Since this group is next to a positively charged ammonium group it prefers to be solvated and this in turn is more favorable than binding with the Zr^4+^.

In contrast, 4HMS presents a rather different situation. Overall, we observe that all four groups are constrained to a preferred orientation which allows for a stable dipole–charge interaction to form between the chelator hydroxamate groups and Zr^4+^. While there are some differences among the angular distributions of different groups, the peaks are localized in two defined regions ranging from 130 to 160° in the Zr^4+^–O–N angles and from 70 to 110° in the Zr^4+^–O–C angles.

### Role of Water in Coordination Chemistry

As mentioned above, the binding mechanism of Zr^4+^ to the chelators in water will naturally involve solvation and desolvation processes. Several of the previous DFT-based calculations have shown by the inclusion of a few coordinating water molecules that specific interactions with the solvent can be an important determinant in binding.^17,21,22^ To understand the role of the solvent, we examined the water coordination around Zr^4+^ for both the DFO and the 4HMS chelation complex.

The Zr^4+^ aquo species has been experimentally determined in acidic aqueous solution by means of an extended X-ray absorption fine structure (EXAFS) and shown to be coordinated with eight water molecules in the first coordination shell, with the bound oxygen atoms located at an average distance of ≃2.2 Å.^53^ Upon binding of the chelate, the number of water molecules changes depending on the number of hydroxamate groups that become coordinated with the ion. To understand the magnitude of this change, we conducted a 200 ns MD simulation of an isolated Zr^4+^ in bulk water. The number of water molecules coordinating Zr^4+^ was then compared to that obtained from the free energy minimum of the chelator complex.

**Figure 7:**
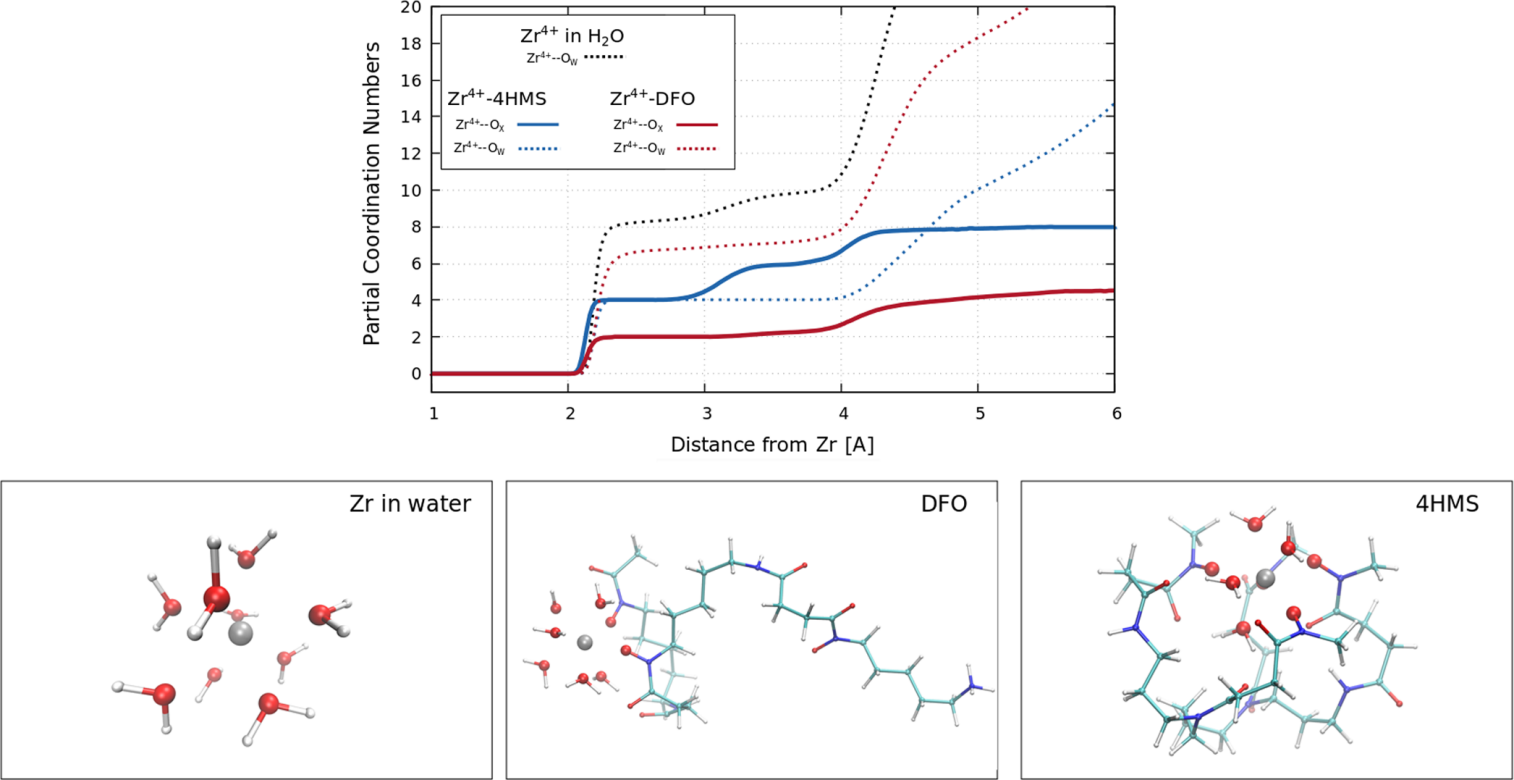
Upper panel: Partial coordination numbers of oxygen atoms around Zr^4+^ as a function of distance from the ion. The dotted black line refers to the simulation of Zr^4+^ in the bulk water. Blue lines refer to the simulation of Zr^4+^–4HMS in water whereby the solid line shows the number of hydroxamate oxygen atoms of 4HMS, and the dotted line shows the number of coordinating water molecules. Red lines refer to the simulation of Zr^4+^–DFO again with the solid line depicting the number of DFO hydroxamate oxygen atoms coordinating Zr^4+^ and the dotted line indicates the number of water molecules. Lower panels: Representative structures from the analyzed simulations, showing only water molecules, whose oxygen atoms lie within a distance of 2.3 Å. Oxygen atoms belonging to the first solvation shell of Zr^4+^ are highlighted with a bigger radius.

In Figure 7, the partial coordination numbers of Zr^4+^ as a function of the distance of oxygen atoms (along with both water molecules and chelators) from the ion are shown. These plots show regions where there are sharp changes in the coordination followed by plateaus which essentially correspond to different solvation shells. In the case of Zr^4+^ aquo species (black line), the number of coordinating water molecules grows to ∼8 between 2.0 and 2.5 Å in agreement with experimental reports^53^ (see a representative snapshot from simulations in the bottom-left panel of Figure 7). As expected, these numbers change for the Zr^4+^–DFO and Zr^4+^–4HMS simulations. In the case of the Zr^4+^–DFO complex, two oxygen atoms from the DFO hydroxamate groups and six oxygen atoms from water are coordinated to Zr^4+^ in the range from 2.2 to 3 Å. Thus, Zr^4+^ remains almost fully solvated when complexed with DFO (see a representative snapshot from simulations in the bottom middle panel of Figure 7).

Interestingly, for the case of Zr^4+^–4HMS at distances less than 3.0 Å, the ion appears to be equally coordinated to four oxygen atoms from 4HMS and four oxygen atoms from surrounding water molecules (see a representative snapshot from simulations bottom-right panel of Figure 7). Similar to DFO, the reduced coordination from the chelator hydroxymate groups likely originates from a balance between enthalpy and entropy leading to differences in the Zr^4+^–O–N and Zr^4+^–O–C angles induced by thermal fluctuations. More specifically, a reduced coordination at the binding site likely enhances the flexibility of the ligand in other parts of the complex. At the same time, this may also reduce the steric strain in both the ligand and solvent. Beyond 3.0 Å, the coordination around the Zr^4+^ increases from 4 to ∼8 originating solely from the oxygen atoms of the hydroxamate groups. Our results, therefore, suggest that the competition between the hydroxamate groups versus specific water molecules in fulfilling a Zr^4+^ coordination sphere can have nontrivial effects in either forming less stable (DFO) or stable complexes (4HMS).

**Figure 8:**
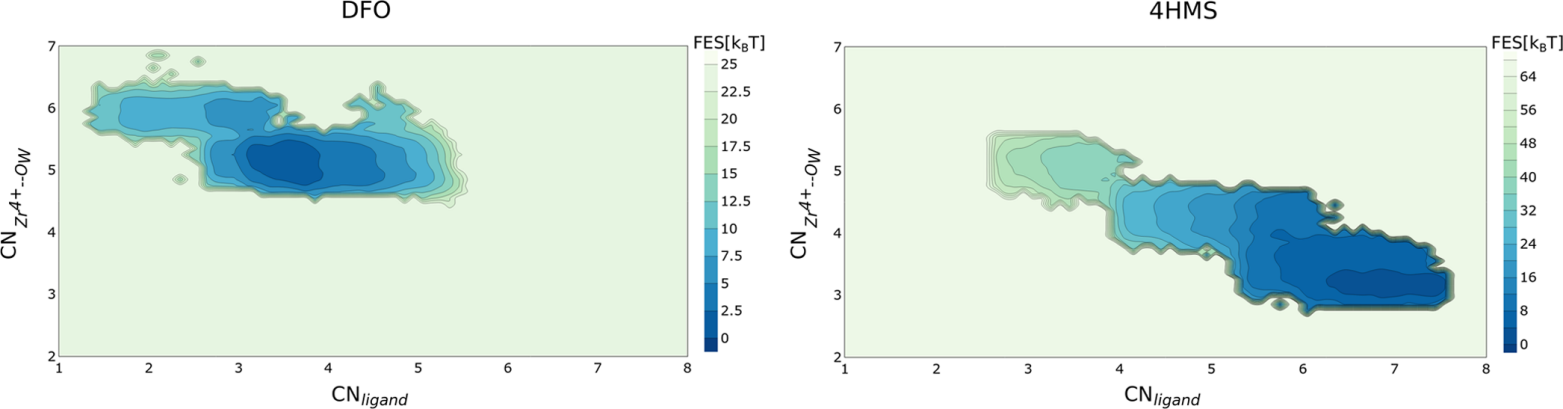
Left panel: Free energy landscape of the Zr^4+^–DFO complex in solvent as a function of the oxygen atoms belonging to the hydroxamate groups coordinating the ligand the ion (*x*-axes) and of oxygen atoms from surrounding water coordinating the ion (*y*-axes). See the Methods section for mathematical definitions of the two collective variables. Right panel: the same for Zr^4+^–4HMS complex.

Given the prominent role of the changes in water solvation around Zr^4+^ upon binding to DFO and 4HMS, it is interesting to understand whether this has any impact on the thermodynamics reported in Figure 5. Specifically, the free energy curves discussed earlier are along one reaction coordinate only, namely, the CN_ligand_ collective variable which counts the oxygen atoms belonging to the chelator hydroxamate groups coordinating the ion. From the one-dimensional umbrella sampling simulations, it is also possible to reconstruct a two-dimensional free energy surface along our original CN_ligand_ variable and another variable that counts the number of water molecules coordinating the Zr^4+^ (labeled as CN_*Zr*^4+^–*O*_*W*__=see the Methods section for definition). Figure 8 shows the two-dimensional free energy surfaces constructed for DFO (left panel) and 4HMS (right panel) as a function of the original CN_ligand_ and of CN_*Zr*^4+^–*O*_*W*__ (on the *y*-axes). For both DFO and 4HMS, we observe that explicit inclusion of solvation in the reaction coordinate does not change our conclusions. For DFO, the free energy minimum involves a structure where there are three to four oxygen atoms of DFO coordinated to Zr^4+^ while for 4HMS, the minimum occurs approximately at 8 hydroxamate oxygen atoms. Furthermore, the free energy differences between the stable and high energy structures are also consistent between the 1d and 2d analyses.

## Discussion

In the past decade, there has been a spurt of research activity in the field of radionuclide science searching for optimized chelating molecules as anchors for radioisotopes in radiopharmaceutical development including Zirconium-89 (^89^Zr). Identifying optimal chelating compounds has enormous potential in radiodiagnostic and radiotherapy applications. One of the most popular chelators that has been the subject of several experimental and theoretical studies is DFO. Despite its wide usage, improving its efficacy in vivo has prompted the design of DFO-like chelators with enhanced coordination with Zirconium. It thus appears timely to harness advanced theoretical tools to guide the design and development of such systems.

The vast majority if not all of theoretical work to date addressing the coordination chemistry of Zirconium and potential chelators has been informed by DFT-based electronic structure calculations. While this approach provides a more accurate treatment of the electronic-induced forces between the nuclei, thermal effects and the role of solvation are neglected. In this work, we have used classical molecular dynamics simulations of Zr^4+^ in water to examine the thermodynamics of the chelating complex for two chelators, namely DFO and 4HMS. Our findings paint a more complex scenario than that observed in previous DFT calculations. Specifically, the DFT-optimized structure in which the three hydroxamate groups of DFO enclose Zr^4+^ is not stable in MD simulations. Free energy calculations instead show that the most stable Zr^4+^–DFO configuration has only two oxygen atoms from DFO coordinated to the cation. Furthermore, water oxygen atoms have a prominent role in filling the ion’s first coordination shell.

For 4HMS, we find that the octacoordinated complex is thermodynamically stable at room temperature and in the presence of solvent molecules with all four hydroxamate groups enclosing the ion. These results highlight the importance of both specific solvation of water molecules and the orientation of the hydroxamate groups relative to the Zr^4+^ in stabilizing the Zr^4+^–4HMS complex. We believe that these molecular insights informed by computational studies offer important design principles that could motivate and guide the experimental search for better Zirconium chelators.

## Computational Methods

For both DFO and 4HMS, we study the case of the deprotonated molecule, where all hydrogens bound to the oxygen atoms of the hydroxamic acid are removed. From experimental acidity constants,^23^ we know that DFO in water at neutral pH has completely protonated hydroxamic groups and the NH_3_^+^ terminal is also protonated. Highly charged Zr^4+^ has a high affinity for hard Lewis bases and complexes stepwise with DFO under deprotonation of the hydroxyl of the hydroxamate group already starting at pH 2. The DFO molecule with a positively charged ammonium group has thus a total charge of −2, whereas the deprotonated 4HMS molecule has a total charge of −4 as it has no –NH_2_ terminus.

The Generalized Amber Force Field (GAFF)^54^ for organic molecules was used to construct interaction potentials for the DFO and 4HMS molecules through the Antechamber package.^55^ The charges are calculated using the AM1-BCC charge model.^56^ The chelator molecules are solvated with 3667 and 3410 water molecules for the DFO and 4HMS molecules, respectively, using the single-point charge (SPC/E) water model.^57^ This water model is rigid body and non-dissociable and therefore speciation events for example between zirconium and water ionic products coming from hydrolysis are not taken into account.^58^ In recent years, Mertz and co-workers have shown that in order to obtain both accurate structural and thermodynamic properties associated with the binding of transition metal ions to proteins, classical force fields can be corrected via the inclusion of an extra attractive term^59^ (), which leads to the following nonbonded interaction potential shown below.
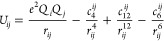
1The extra term () mimics charge-induced dipole interactions. The force field parameters for Zr^4+^ ion are taken from ref (59).

All of the MD simulations are run using the Amber2020 package. The Zr^4+^–chelate complexes are placed in a cubic box of water where the closest distance between the complex and the box wall was 17 Å. In the case of the DFO, two chloride ions are added to neutralize the system. For both systems, we conducted an equilibration procedure before moving to production runs. First, a minimization step is performed, then the temperature is gradually brought from 0 to 300 K along an NVT simulation of 20 ps, and finally, the density is equilibrated through a simulation in the NPT ensemble for 2 ns using a Berendsen barostat^60^ fixed at 1 bar. This equilibration phase is followed by a 1μs simulation in the NVT ensemble at a temperature of 300 K. The box sizes for DFO along the *x*, *y*, and *z* are 59.9, 51.7, and 46.9 Å, respectively, while for 4HMS, it is 53.8, 53.1, and 48.1 Å. In all of the MD simulations, the time step is set to 2 fs where the temperature is controlled using a Langevin thermostat^61^ using a time constant of γ = 2 ps^–1^. A cutoff of 11 Å is used for the nonbonded interactions. Long-range corrections to the van der Waals are included. The particle mesh ewald (PME)^62^ is used to treat the long-range part of the Coulomb interactions.

To study the ion-chelator coordination chemistry, we choose as a collective variable the coordination number between Zr^4+^ and the oxygen atoms belonging to the hydroxamate groups of the chelator defined as
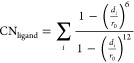
2

The index *i* runs over the oxygen atoms belonging to the hydroxamate groups of the chelator (6 in the DFO, 8 in the 4HMS), *d_i_* is the distance between *i*-oxygen and Zr^4+^, *r*_0_ = 5 Å is the cutoff parameter of the switching function. As described in the Results Section, we used the Umbrella Sampling technique^25^ to reconstruct the free energy landscape of the Zr^4+^–DFO and Zr^4+^–4HMS complex in solvent as a function of the CN_ligand_. These constrained simulations were obtained using the Amber suite combined with tools from PLUMED open source library.^63^ The free energy landscapes are then reconstructed using the WHAM method (code from ref (30)).

To study the ion–water coordination chemistry, we defined a second collective variable that counts the number of oxygen atoms from surrounding water molecules coordinating Zr^4+^
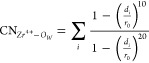
3The index *i* runs over the oxygen atoms belonging to all water molecules, *d*_*i*_ is the distance between *i*-oxygen and Zr^4+^, and *r*_0_ = 2.5 Å is the cutoff parameter of the switching function. The two-dimensional free energy landscapes are also obtained using the WHAM method (code from ref (30)).
